# Health and survival of young children in southern Tanzania

**DOI:** 10.1186/1471-2458-8-194

**Published:** 2008-06-03

**Authors:** Joanna RM Armstrong Schellenberg, Mwifadhi Mrisho, Fatuma Manzi, Kizito Shirima, Conrad Mbuya, Adiel K Mushi, Sosthenes Charles Ketende, Pedro L Alonso, Hassan Mshinda, Marcel Tanner, David Schellenberg

**Affiliations:** 1 Ifakara Health Research & Development Centre, Ifakara, Tanzania; 2 Department of Infectious and Tropical Disease, London School of Hygiene & Tropical Medicine, London, UK; 3 Ministry of Health, Tanzania; 4 Hospital Clinic I Provincial, Barcelona, Spain; 5 Swiss Tropical Institute, Basel, Switzerland

## Abstract

**Background:**

With a view to developing health systems strategies to improve reach to high-risk groups, we present information on health and survival from household and health facility perspectives in five districts of southern Tanzania.

**Methods:**

We documented availability of health workers, vaccines, drugs, supplies and services essential for child health through a survey of all health facilities in the area. We did a representative cluster sample survey of 21,600 households using a modular questionnaire including household assets, birth histories, and antenatal care in currently pregnant women. In a subsample of households we asked about health of all children under two years, including breastfeeding, mosquito net use, vaccination, vitamin A, and care-seeking for recent illness, and measured haemoglobin and malaria parasitaemia.

**Results:**

In the health facility survey, a prescriber or nurse was present on the day of the survey in about 40% of 114 dispensaries. Less than half of health facilities had all seven 'essential oral treatments', and water was available in only 22%. In the household survey, antenatal attendance (88%) and DPT-HepB3 vaccine coverage in children (81%) were high. Neonatal and infant mortality were 43.2 and 76.4 per 1000 live births respectively. Infant mortality was 40% higher for teenage mothers than older women (RR 1.4, 95% confidence interval (CI) 1.1 – 1.7), and 20% higher for mothers with no formal education than those who had been to school (RR 1.2, CI 1.0 – 1.4). The benefits of education on survival were apparently restricted to post-neonatal infants. There was no evidence of inequality in infant mortality by socio-economic status. Vaccine coverage, net use, anaemia and parasitaemia were inequitable: the least poor had a consistent advantage over children from the poorest families. Infant mortality was higher in families living over 5 km from their nearest health facility compared to those living closer (RR 1.25, CI 1.0 – 1.5): 75% of households live within this distance.

**Conclusion:**

Relatively short distances to health facilities, high antenatal and vaccine coverage show that peripheral health facilities have huge potential to make a difference to health and survival at household level in rural Tanzania, even with current human resources.

## Background

More than ten million children under five years die every year [1]. Most of these deaths are in developing countries and roughly two-thirds could be prevented by interventions that are already available. The leading causes of these deaths are malaria, pneumonia, respiratory infections and deaths during the neonatal period due to pre-term birth, infections, and birth asphyxia. Malnutrition is the most common underlying cause of child deaths. These hard facts continue to shock, and have led to calls for action to prevent child deaths and reduce inequities in child survival [2,3]. The Millennium Development Goal (MDG) for child survival is intended to encourage national governments to focus both policies and finances on child health issues, but progress in sub-Saharan Africa is trailing behind that in other parts of the world [4].

Why are life-saving interventions not reaching poor mothers and children? Interventions need a delivery system and health systems are often weakest where child mortality is highest [5]. New interventions and initiatives are often developed with little attention given to how they would be delivered, particularly to the poorest or most marginalised groups, and with relatively little investment directed at the health system itself [6,7]. An analysis of current funding priorities for research to address the leading causes of death in children showed that the National Institutes of Health and the Bill and Melinda Gates Foundation allocated 97% of funding for research to develop better technologies and just 3% for delivering interventions to those who need them [8].

Although it is no great surprise that poorer children are more likely than their better-off peers to be exposed to health risks [9], action to redress these inequities has been slow. Not only does poverty lead to conditions that increase exposure and reduce resistance to infection, but also poorer children tend to have worse care-seeking for both curative and preventive services than those who are better-off; and the chances of getting the preventive and curative interventions they need are also worse [6]. The "equity lens" approach of analyzing child health indicators by socio-economic status, sex or ethnicity, to look at inequalities in child health issues, can reveal gaps in coverage in certain groups that cannot be redressed without policy change [10,11]. In particular, universal coverage is only possible if the poorest children are reached.

Tanzania, with a population over 36 million people, is one of the poorest countries in the world [12]. Recent trends in infant survival are encouraging, with a drop in infant mortality rate (IMR) from 99 in 1994–9 to 68 in 2000–2004 [13]. National figures, however, obscure local variations: in the 20 regions of the country infant mortality varies widely with estimates for 1999 ranging from 41 per 1000 live births for Arusha Region to 129 for Lindi Region in 1999 [12]. The health system has broader reach than in many sub-Saharan African countries, with one health facility for every 9,000 people. Health sector and local government reform is ongoing, and local councils have increased autonomy and control over their own health budgets and plans. The Ministry of Health and development partners (at the time of this study, the World Bank and the Governments of Denmark, Ireland, the Netherlands, Norway, Switzerland and the UK) pool resources in a common "basket" from which funds are then directly disbursed to districts through special accounts of the Council Health Management Teams. A limited amount of this donor-supported "basket" funding from the health Sector-Wide Approach is therefore available for local councils to implement their own plans. Nationally, spending on health is estimated to be $11.34 per capita, of which almost half is contributed by households [14].

In Tanzania, as in many developing countries, vital registration and routine health information are incomplete. Cross-sectional household surveys are often used to estimate levels and trends in mortality, morbidity and intervention coverage from the community perspective. More rarely, cross-sectional health facility surveys are used to assess aspects of the structure and function of the health system, including quality of care. As an example, consider a poor rural family who happen to live close to a peripheral dispensary. They may take their children to this clinic for vaccination, but unless effective drugs and trained well-motivated staff are also there when they are sick, they will not receive the interventions they need. Large-scale linked data from both household and health facility perspectives provide an opportunity to study the health delivery system in parallel with health and social issues at community level.

The aim of this paper is to provide a comprehensive description of a rural malaria endemic area, including the health systems context, in which integrated malaria control strategies can be implemented and tested for community effectiveness and equity effectiveness. We present information on health in children under two years and on infant survival from both household and health facility perspectives, using information from a 21,000-household survey in five districts of southern Tanzania and a census of health facilities serving this population. We assess evidence of inequalities by sex, ethnic group, socio-economic status and distance to health service providers. Describing differentials in intervention coverage and infant mortality is important because reducing inequalities in health has been singled out as the major challenge to be addressed in order to improve global health [15]. Of particular relevance is the finding that interventions for promoting child health may in fact increase inequalities before they reduce them [16]. The surveys were part of the baseline work of an effectiveness study of Intermittent Preventive Treatment for malaria in infants (IPTi), within the IPTi Consortium [17].

## Methods

### Study area

**Figure 1:**
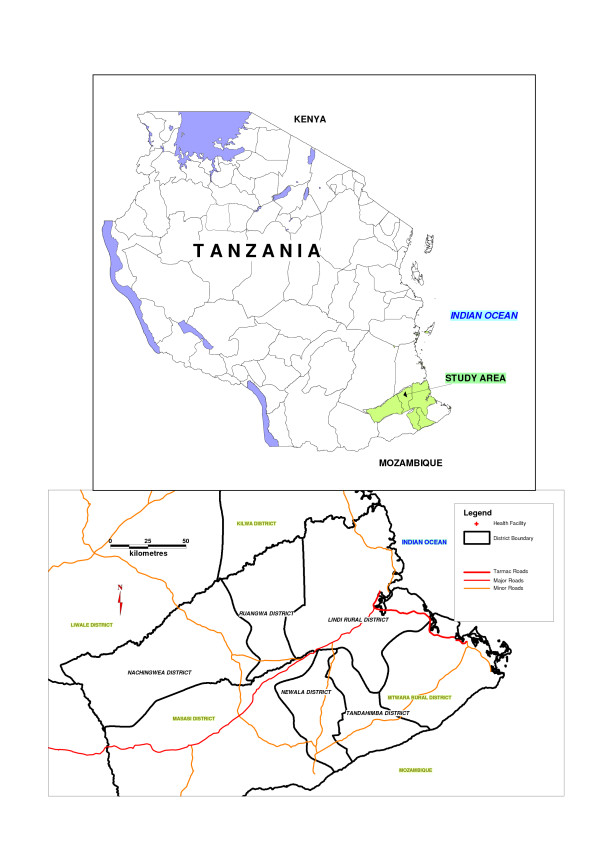
Map showing the location of the study area within Tanzania (top) and the five districts comprising the study area (bottom).

Nachingwea, Lindi Rural, Ruangwa, Tandahimba and Newala Districts are in Southern Tanzania and had a total population of about 900,000 people in 2002 (Figure 1) [12]. These districts are sub-divided into administrative areas called divisions, with 3 to 10 divisions in each district and a total of 24 divisions in the study area. Parts of Tandahimba and Newala are on the Makonde Plateau, up to 900 m above sea level. Lindi Rural, Ruangwa and Nachingwea have hilly areas as well as low lying plains. The major permanent rivers in the region are the Lukuledi, Matandu, Mbwemkulu and Mavuji. There are two main rainy seasons, November to December and February to May, but rain is not uncommon in any month. Malaria is endemic and transmission occurs all year round. The area had a wide mix of ethnic groups, including the Makonde, Mwera, Yao and many others. Although most people speak the language of their own ethnic group, Swahili is also widely spoken. The most common occupations are subsistence farming, fishing and small scale trading. Cashew nuts, sesame and groundnuts are the major cash crops while food crops are cassava, maize, sorghum and rice. Most people live in mud-walled and thatched-roof houses: a few houses have corrugated iron roofs. Common water supplies are hand-dug wells which rely on seasonal rain, communal boreholes, natural springs and river water. Most rural roads are unpaved: some are not passable during rainy seasons while others are too steep for vehicles to pass. The public health system comprises a network of dispensaries, health centres and hospitals offering a varying quality of care. Not all health facilities have a qualified prescriber (Medical Officer, Assistant Medical Officer, Clinical Officer or Assistant Clinical Officer) and sick children are not infrequently managed by nursing cadres (Nursing Officers, Nurse Midwives, Public Health Nurse 'B' or Maternal and Child Health Aides) even though these staff are, strictly speaking, not supposed to prescribe. Staff in these nursing cadres are generally responsible for preventive services such as antenatal care and well-child visits for weighing and vaccination. With the exception of Lindi Rural, each district has a District hospital. Some villages have village health workers. Children under five years of age are exempted from paying fees at any government health facility. In the five years prior to the 2002 National Census, the infant mortality rate estimated by indirect methods (probability of dying by the first birthday) was 129/1000 and 126/1000 in Lindi and Mtwara Regions respectively.

### Health Facility Survey

We did a survey in all 134 health facilities in the area in October 2004, including hospitals, health centres and dispensaries of the public health care delivery system, non-governmental not-for-profit organizations and the private sector. The overall focus was the availability and accessibility of maternal and child health care. Our aim was to undertake a structural and functional assessment by documenting the availability of vaccines, drugs, supplies and services essential for child health. We interviewed health workers, checked availability and functioning of equipment, and checked drug and vaccine stocks. The survey was conducted using an adapted World Health Organisation (WHO) health facility survey tool [18], without case-management observation or exit interviews. The tool included modules on health services, equipment and supplies. The survey was done by 16 interviewers working in groups of two, forming eight teams, with two supervisors who assisted the survey co-ordinator. Training was carried out over a period of five days and included interview technique, group work, role-plays and practical fieldwork including a pilot-test of the survey instruments. A letter of introduction from each Council Health Management Team, signed by the District Medical Officer and the District Executive Director, was given out at each facility before the interviews. To help assure the quality of data, at least one interview was accompanied by a supervisor each day. All forms completed each day were reviewed in the evening and feedback given to the interview teams.

### Household Survey

We did a cross-sectional survey in a representative cluster sample of households from the five districts in July–August, 2004. Thirty clusters of 30 households were sampled from each division, giving a total of 720 clusters and approximately 21,600 households. Within any division, every household had an equal chance of being selected. The number of clusters selected from each ward (an administrative sub-unit of a division) was determined by probability proportional to the size of the ward, on the basis of the 2002 National Census population figures. The required number of clusters was then selected at random from a list of all sub-villages (*vitongoji*, singular *kitongoji*), as supplied by the district council. Within each sub-village, 30 households were selected at random using a modified Expanded Programme of Immunisation (EPI) – type sampling scheme that ensured an equal chance of any household being selected. Since there was no list of all households in the kitongoji, the supervisor went to the centre of the kitongoji and threw a pen to choose a random direction. He or she walked in the direction indicated until he reached the edge of the kitongoji, sketching a map of all the households passed, and numbering them as he went. For this purpose a household is a group of people who eat from the same cooking pot. One of these houses was selected at random as the starting point, or house 1 of the cluster. Throwing a pen to choose a random direction, the supervisor walked in that direction until he came to another household, which was the second house of the cluster, and so on. If there was a junction in the path, a pen was thrown again to select from the choices available. This procedure was repeated until 30 households were counted. All households were selected for an interview regardless of whether there were any women or children. Villages were visited one day before the survey interviewers arrived, and an invitation letter left in each of the selected households. Both village and kitongoji leaders were briefed about the visit. Interviewers went from house to house, and if there was nobody at home an interviewer returned later in the day. No substitute household was included if the owner was repeatedly absent or did not wish to participate. The supervisor visited all households where people were reportedly absent or did not wish to take part, as a quality control measure.

We administered a modular questionnaire using handheld computers (Personal Digital Assistant or PDA)[19]. A household module included information on who lived in the household, dates of birth, education, and occupation, and ethnic group of the household head. We collected information on proxy markers of household socioeconomic status including ownership of a radio, a bicycle, animals, and poultry, whether the house had a corrugated iron roof, whether the house was owner-occupied or rented, and whether it was connected to the mains electricity supply. We also recorded the location of the household using a handheld Global Positioning System (GPS). In a birth history module, similar to that used in the Demographic and Health Surveys, we obtained information on survival of all children born in the five years prior to the survey with a view to getting complete and accurate information for the three years prior to the survey, July 2001 – June 2004. Mothers were asked about dates of birth, the sex of the baby, whether they were twins or singletons, and whether the child was still alive. For those who had died, we asked for the date of death. We also asked about antenatal care as well as the use of sulphadoxine-pyrimethamine (SP) for Intermittent Preventive Treatment for malaria in the most recent completed pregnancy (IPTp), and about mosquito net use for women who reported being pregnant at the time of the interview. For a sub-group of 8 in every 30 clusters (27% of households), stratified on division, we also included modules about the health of all children under two years. Mothers or carers (here we use the term 'mother' to denote the main carer) of all children under two years were interviewed about whether or not the child was currently breastfed and if so what other food or drink the child had received over the previous 24 hours. Information on routine vaccinations was documented directly from health cards or other written records. Where no such record was available we asked about the number of doses and type of vaccines received, but we did not ask for dates of each dose. It should be noted that estimates of vaccine coverage reported here are based on written information alone and are likely to be conservative. Mothers were asked whether the child had received vitamin A supplementation, and if so, how many months ago. Mothers were then asked about use of mosquito nets and their treatment, the name and location of their nearest health facility, and about any illness each child had during the 2 weeks prior to the survey, and what action had been taken. For children who had been sick, we asked about utilisation of appropriate (non-traditional) health care providers including village health workers, dispensaries, health centres, hospitals, or private doctors. Children under 2 years were invited to attend a measuring station set up in the middle of the village where they were weighed using hanging scales (Salter, UK). A finger-prick sample of blood was collected and haemoglobin (Hb) concentration measured using a battery-powered Hemocue photometer (HemoCue AB, Angelholm, Sweden). We tested for *Plasmodium falciparum *malaria parasitaemia using the Paracheck rapid diagnostic test device (Orchid Biomedical Systems, Goa, India). Children found to be anaemic (Hb < 11 g/dl) or parasitaemic were offered treatment according to Tanzanian national guidelines (14 day course of ferrous sulphate, SP, or both). Children with Hb < 5 g/dL were encouraged to attend a hospital urgently and their transportation facilitated.

The survey was carried out by 91 interviewers in 13 teams of 7, each with a supervisor, a driver and a car. Interviewers were trained for two weeks. All teams worked in all divisions. The training included interview technique and probing for dates using local event calendars. A detailed field manual was prepared, piloted during the training sessions and carried in the field by all interviewers. The trainees included participants from all study districts. At the end of the training period a pilot survey was carried out in part of Mtwara Rural district. During the survey, at least one interview was accompanied by a supervisor each day and he/she also repeated key aspects of further interviews independently at random. In these repeat interviews, the head of household and one woman aged 15–49 were asked a subset of questions. Any discrepancies between the initial and repeat interviews were discussed with the interviewer and appropriate action taken.

### Data processing and analytical methods

For the health facility survey, data were collected using conventional paper forms. Double data entry was done using DMSys software for clinical trials (SigmaSoft International), with range and consistency checks on entry and discrepancies between the two versions resolved with reference to the original forms. Data were summarised using tables. For the household survey, all data were entered into PDAs at the point of data collection. Standard range, consistency and completeness checks were carried out at that time. Particular attention was paid to the accuracy of dates of vital events because of the rapid change in the risk of death within the first year of life and the fact that age is typically prone to errors of recall. Analysis of household data was done in Stata (version 8, College Station, Texas, USA) in accordance with the design of the survey, using "svy" commands such as svytab to summarise and adjust for the clustered nature of the data. We created an index of household wealth based on ownership of assets. The household wealth index was the weighted sum of household characteristics including whether they owned the house they occupied, whether they owned consumer durables such as a bicycle, radio, or tin roof, whether they owned animals or poultry, whether they used wood for cooking, and whether they had mains electricity. The weights for the assets in the index were generated by Principal Components Analysis (PCA) on the correlation matrix [20]. Households were categorized into one of five equal sized groups from the most poor to the least poor. P-values for testing hypotheses concerning inequalities by sex, ethnic group and distance from the health facility were from design-based F-tests, equivalent to ordinary chi-squared tests adjusted for clustering. P-values for testing hypotheses concerning inequalities by socio-economic status quintile were based on a test for trend with socio-economic status from a logistic regression model adjusted for clustering. Mortality rates were derived from the birth histories from all births between July 2001 and June 2004, with time at risk calculated from the date of birth until the date of the first birthday or the end of June 2004, whichever was the earliest, or the date of death for those who died before their first birthday. Mortality rates were modelled using Poisson regression with robust standard errors to adjust for clustering. Weight for age Z-scores were calculated with reference to the US National Centers for Health Statistics standards using the EPINUT module of EPI-Info (CDC Atlanta, Georgia, US). Underweight was defined as weight-for-age z-score of less than -2, excluding outliers (z-score of < -5 or > 5). GPS data were cleaned by removing outliers in each cluster (households more than 10 km from the median location of households in that cluster) and straight-line distance to the nearest health facility was calculated, then categorised as over or under 5 km, which is roughly one hour's journey on foot.

### Ethical approval

The study was undertaken within the framework of the assessment of the community effectiveness of IPTi, part of the IPTi Consortium [17]. We received ethical approval from the local and national institutional review boards (Ifakara Health Research and Development Centre, Ifakara, and the National Tanzania Medical Research Co-coordinating Committee) through the Tanzania Commission for Science and Technology. In addition ethical and research clearance was also obtained from institutional review board of the London School of Hygiene and Tropical Medicine, UK, and Ethics Commission of the Cantons of Basel-Stadt and Basel-Land, Switzerland. During field work, information sheets in Swahili about the study were given out, explaining why it was being done, by whom, and what it would involve. In the household survey, written consent of all household heads was sought. Confidentiality of all study participants was assured.

## Results

During the household survey in July and August 2004 we visited 21,474 households in 720 clusters. In 497 households (2%) no-one was present to give consent and in a further 91 (0.4%) the household head was not willing to participate. Characteristics of the remaining 20,886 households are shown in Table 1. Mean household size was 3.9 people and one-fifth of all households had more than six residents. Makonde and Mwera were the most common ethnic groups, with over 80% of all household heads coming from these two groups. The majority of household heads were male (76%). The median distance to the nearest health facility was 3.2 km with an inter-quartile range from 0.8 km to 5.2 km.

**Table 1:** Distribution of the households and women studied: demographic, outcome of pregnancy and antenatal care (source: household survey)

**Characteristic**	**Category**	**Number**	**Percentage**
Household size	1–3	9,396	45%
	4–5	7,346	35%
	6 and over	4,144	20%
Ethnic group of household head*	Makonde	8,828	42%
	Mwera	8,518	41%
	Yao	1,009	5%
	Other	2,530	12%
Sex of household head	Male	15,794	76%
	Female	5,092	24%
Distance to nearest health facility**	Under 5 km	14,183	71%
	5 km and over	5,712	29%
Women aged 15–49 years	Total resident	19,935	
	Interviewed	19,007	95%
	Gave birth in previous 3 years	7,413	39%
Experience of at least one child (under 5 years) death	Yes	7,157	38%
	No	11,850	62%
Place of delivery for the most recent birth	Health Facility	4,029	39%
	Elsewhere	6,255	61%
Antenatal clinic use in most recent pregnancy^§^	Yes	2,431	88%
	No	345	12%
IPTp in most recent pregnancy^§^	Yes	1,662	60%
	No	1,114	40%
Net last night in current pregnancy^§§^	Yes	271	25%
	No	800	75%
Ever-treated net last night in current pregnancy^§§^	Yes	92	9%
	No	979	91%

* Missing for 1 household

** Missing for 991 households (5%)

^§ ^For pregnancies in the year before the survey, excludes current pregnancies

^§§ ^Missing for 2 women

We attempted to interview all 19,935 women aged 15–49 years living in these households (Table 1). About one-quarter had no formal education (28%), but of those who had been to school most had completed primary education (73%). We did a birth history interview with 19,007 (95%) of the women, and found that 38% had experienced at least one child death. Only 39% of the most recent births took place in a health facility, with the majority taking place at home. Nevertheless, antenatal clinic attendance was very common, with 88% of women having attended clinic during their most recent (completed) pregnancy. Sixty percent of the women said they had taken one or more doses of antimalarials as Intermittent Preventive Treatment of malaria in pregnancy (IPTp), although only 42% recalled that they had been given sulphadoxine-pyrimethamine (SP) as IPTp. We asked women who said they were pregnant at the time of the survey whether they had slept under a mosquito net the previous night, and if so whether it was treated. One-quarter of pregnant women (25%) said they had used a net the previous night and just 9% had used a net ever treated with insecticide.

A summary of the assets owned by households in each quintile of the socio-economic status score from principal components analysis is shown in Table 2. Typically, a household in the poorest quintile would have no bicycle, radio, or poultry, and would have a thatched roof. A typical household in the least poor quintile would own a bicycle and radio, a few chickens or ducks, and a tin roof.

**Table 2:** Asset ownership for households in each socio-economic status quintile (source: household survey)

				Household "assets": percentage with....							
Socio-economic status quintile	Number of households	Percentage of households	Mean SES score	Rent the house they occupy	Bicycle	Radio	Use wood for cooking	Animals	Poultry	Mains electricity	Tin roof

Most poor	4,419	21%	-1.354	0%	0%	0%	100%	0%	9%	0%	0%
Very poor	4,622	22%	-0.875	0%	13%	21%	100%	11%	62%	0%	0%
Poor	3,341	16%	-0.272	9%	30%	37%	100%	13%	75%	0%	21%
Less poor	4,121	20%	0.486	6%	72%	74%	99%	18%	65%	0%	21%
Least poor	4,125	20%	2.164	15%	83%	87%	82%	37%	78%	4%	70%

**All**	**20,628**	**100%**		**6**%	**39**%	**43**%	**96**%	**16**%	**57**%	**1**%	**22**%

			*Factor loadings*	*0.22*	*0.42*	*0.43*	*0.38*	*0.26*	*0.28*	*0.32*	*0.45*

Availability of staff, vaccines and drugs on a single day in October 2004 in the health facilities serving this population is shown in Table 3. Of 118 facilities supplying vaccination services, 77% had Bacille Calmette-Guerrin (BCG), Diptheria-Pertussis-Tetanus-Hepatitis B (DPT-HepB), oral polio vaccine (OPV), measles and tetanus toxoid (TT) vaccine in stock and almost all (97%) had a fridge. However, as a rough indication of how effective the fridges were, only about half of all facilities had a fridge that would freeze water (54%). Most of the 134 facilities providing treatment services had oral rehydration solution (ORS) (93%) and SP (93%) in stock and slightly fewer had co-trimoxazole (73%). Less than half (42%,) had all seven 'essential oral treatments' available, ie. ORS, SP, co-trimaxazole, vitamin A, ferrous sulphate, paracetamol and mebendazole. Water was only available in 22% of the facilities. Injectable treatment for pre-referral care was available in roughly half of both dispensaries and health centres (52% and 43% respectively). We assessed human resources at health centres and dispensaries in terms of qualified prescribers and nurses. Only three-quarters of dispensaries had at least one prescriber and a similar proportion had at least one nurse (75% and 76% respectively). Furthermore, absenteeism was common in both nursing and prescribing cadres: only about 40% of dispensaries had a prescriber or a nurse present on the day of the survey (41% and 43% respectively). The most common reasons for absence were official travel (including meetings and seminars), leave and long-term training. Most facilities had received a supervision visit in the 6 months prior to the survey (84%, 112/134) but only a few recalled supervision visits including observation of case-management (17%, 23/134). (It should be noted that some under-reporting of this issue is possible: on a few occasions the interviewee was not the person in-charge of the facility and they may not have been present during all supervision visits.) Transport for referral care was rarely available, either by bicycle or ambulance (16%, 21/134), and one-quarter of those in-charge of a health facility said that they had wanted to refer a sick child at some point but had been unable to (25%, 34/134).

**Table 3:** Availability of staff, vaccines and drugs in health facilities on a single day in October 2004 (source: health facility survey)

**Characteristic**		**Categories**	**Number of facilities**	**% of facilities**	95% CI*
VACCINES AND EQUIPMENT FOR VACCINATION (in all 118 facilities supplying vaccination services)					

All essential vaccines available (BCG, DPT-HepB, OPV, Measles, TT)			91/118	77%	68,84
Fridge		Available	115/118	97%	93,99
		Available & freezes	64/118	54%	45,63

ORAL AND INJECTABLE DRUGS (in all 134 facilities)					

Essential oral treatments available (ORS, SP, co-trimoxazole, vitamin A, ferrous sulphate, paracetamol, mebendazole) (SP was the first-line antimalarial drug at the time of the survey)		All available	57/134	42%	34,51
		ORS	125/134	93%	88,97
		SP	124/134	93%	87,96
		Co-trimoxazole	98/134	73%	65,80
Water available			30/134	22%	16,30
Injectable treatment for pre-referral care (quinine, gentamicin or ampicillin or chloramphenicol, benzylpenicillin or cristapen or PPF (procaine penillin))		In dispensaries	59/113	52%	43,62
		In health centres	6/14	43%	18,71

HUMAN RESOURCES (in all 127 health centres & dispensaries)					

Prescribers (Medical Officers, Assistant Medical Officer, Clinical Officer or Assistant Clinical Officer)	Dispensaries	Present	46/113	41%	32,50
		Employed	85/113	75%	66,83
	Health Centres	Present	8/14	57%	29,82
		Employed	12/14	86%	57,98
Nurses (Nursing Officer, Nurse Midwife, Public Health Nurse 'B' or Maternal and Child Health Aides)	Dispensaries	Present	49/113	43%	34,53
		Employed	86/113	76%	67,84
	Health Centres	Present	11/14	79%	49,95
		Employed	13/14	93%	66,100

SUPERVISION & REFERRAL (in all 134 facilities)					

At least one supervision visit in previous 6 months			112/134	84%	76,89
At least one supervision involving case-management observation in previous 6 months			23/134	17%	11,25
Transport for referral (bicycle or ambulance)			21/134	16%	10,23
Has the in-charge ever wanted to refer a sick child but been unable to?			34/134	25%	18,34

*Assuming binomial distribution

During the household survey we interviewed mothers of all 1,414 children under two years about preventive care and recent illness in a randomly selected sub-group of 27% of households (192 clusters out of 720). Vaccine coverage was high, with 80–90% of all children aged 12–23 months having received BCG, three doses of DPT, and at least three doses of OPV before one year of age (Table 4). Measles vaccine coverage was slightly lower at 69%. Almost one-third of children had slept under a mosquito net the night before the survey (30%) but most of these nets were untreated. This is a conservative estimate because net use is likely to be a few percentage points higher in the rainy season, when mosquito densities are higher (unpublished observations). Only about one in ten children used a net that had ever been treated with insecticide (11%) or one which had been treated in the last year (9%). Malaria parasitaemia due to *P falciparum *was found in 62% of children under two years and severe anaemia (Hb < 8 g/dL) in 31%. Almost half of all children had been ill in the two weeks before the survey (48%), and of those who had been ill almost half had sought care from a so-called "Western-style" care provider such as a hospital, health centre, or dispensary (46%). Roughly one in every 6 children had been admitted to a health centre or hospital for care in the year before the survey (16%), with malaria being the most common reported primary cause (52%) followed by diarrhoea (15%), pneumonia and anaemia (11% each). Exclusive breastfeeding in children under six months of age was reported in only one-quarter of children (24%). Roughly one-third of all under-two-year-olds were underweight, with a weight-for-age z-score of minus 2 or lower (32%).

**Table 4:** Preventive care and recent illness in children under two years (source: household survey)

Indicator	Number of children	Percentage of children
VACCINE COVERAGE & VITAMIN A (in children aged 12–23 months)		

BCG before 12 months of age	647/724	89%
DPT-HepB3 before 12 months of age	588/724	81%
OPV3 before 12 months of age	657/724	91%
Measles before 12 months of age	496/724	69%
Vitamin A in previous 6 months	500/710	70%

MOSQUITO NETS (in children aged 0–23 months)		

Slept under a net last night	417/1,401	30%
Slept under a treated net last night	159/1,401	11%
Slept under a recently-treated net last night	125/1,401	9%

MALARIA AND ANAEMIA (in children aged 0–23 months)		

Malaria parasitaemia	780/1,254	62%
Severe anaemia (Hb < 8 g/dL)	384/1,256	31%

RECENT ILLNESS AND CARE-SEEKING (in children aged 0–23 months)		

Illness in the last two weeks	674/1,410	48%
Sought care from a Western-style health care provider (hospital, health centre, dispensary etc)	309/674	46%
Admissions in the previous year	227/1,414	16%

NUTRITION		

Exclusive breastfeeding in children under 6 months old	79/332	24%
Underweight (weight-for-age z-score under -2) in children 0–23 months old	400/1,243	32%

We looked for inequalities in indicators of preventive care and recent illness by sex, ethnic group of the household head, household socio-economic status and distance from health facilities. We found little evidence of inequalities by sex, although admissions in the previous year were slightly more common in boys than girls (19% vs 13%, p = 0.008, Table 5). Children living in Makonde-headed households were less likely to be anaemic and to be underweight than those in other groups (Table 6: 25% vs 34%–37% for severe anaemia, p = 0.007; 29% vs 37% for underweight, p = 0.04). Coverage of all vaccines was 10% to 20% lower in the poorest households than in the least poor, with the trend of lower coverage in poorer children reaching statistical significance for DPT-HepB3 and measles vaccine (Table 7: ratio of poorest to least poor 0.8 – 0.9, p for trend < 0.05). Mosquito net use was 70% to 80% lower in the poorest children than in the least poor, for both treated and untreated nets (ratio of poorest to least poor 0.2 – 0.3, p for trend < 0.0001). Malaria parasitaemia was more common in the poorest children compared to the least poor (68% in poorest compared with 50% in least poor, ratio 1.4, p for trend < 0.0001). There was an even more marked inequality for severe anaemia (Hb < 8 g/dL), which was found in 46% of the poorest children but only in 21% of the least poor (ratio 2.2, p for trend < 0.0001). In contrast, there was little evidence of inequality in recent illness, care-seeking or admissions in the previous year by socio-economic status. Exclusive breastfeeding in children under 6 months was almost twice as common in the poorest children compared to the least poor, with the trend not quite reaching statistical significance (p for trend = 0.09, ratio poorest to least poor 1.8). Underweight (weight-for-age z-score < -2) was 1.7 times more common in the poorest children compared to the least poor (P for trend = 0.001). Inequalities by distance from the nearest health facility were also apparent: coverage of all vaccines was 5 to 11 percentage points lower in households further than 5 km from their nearest facility than in those living closer (Table 8: p < 0.05). Mosquito net use was also more common in children living closer to health facilities (33% for those living under 5 km away and 25% in others: p = 0.05). Although illness in the last two weeks was equally common in those living nearer and further from a health facility, appropriate care-seeking was more common in those living closer to health facilities (51% and 37%, p = 0.002). Despite this, admissions in the previous year showed no disparity by distance from the nearest health facility (15% and 14%, p = 0.62).

**Table 5:** Inequalities by sex in preventive care and illness in children under two years (source: household survey)

**Indicator**	**Number of children**	**Percentage of children**		**P**
			
		**Boys**	**Girls**	
**VACCINE COVERAGE & VITAMIN A **(in children aged 12–23 months)				

BCG before 12 months of age	647/724	88%	91%	0.17
DPT-HepB3 before 12 months of age	588/724	82%	80%	0.37
OPV3 before 12 months of age	657/724	90%	92%	0.40
Measles before 12 months of age	496/724	66%	71%	0.19
Vitamin A in previous 6 months	500/710	71%	70%	0.87

**MOSQUITO NETS **(in children aged 0–23 months)				

Slept under a net last night	417/1,401	32%	28%	0.11
Slept under a treated net last night	159/1,401	13%	10%	0.19
Slept under a recently-treated net last night	125/1,401	11%	7%	0.03

**MALARIA AND ANAEMIA **(in children aged 0–23 months)				

Malaria parasitaemia	780/1,254	62%	62%	0.90
Severe anaemia (Hb < 8 g/dL)	384/1,256	67%	72%	0.07

**RECENT ILLNESS AND CARE-SEEKING **(in children aged 0–23 months)				

Illness in the last two weeks	674/1,410	49%	47%	0.53
Sought care from a Western-style health care provider (hospital, health centre, dispensary etc)	309/674	44%	48%	0.28
Admissions in the previous year	227/1,414	19%	13%	0.008

**NUTRITION**				

Exclusive breastfeeding in children under 6 months old	79/332	23%	25%	0.68
Underweight (weight-for-age z-score under -2) in children 0–23 months old	400/1,243	34%	31%	0.32

**Table 6:** Inequalities by ethnic group in preventive care and illness in children under two years (source: household survey)

**Indicator**	**Number of children**	**Percentage of children**				**P**
			
		**Makonde**	**Mwera**	**Yao**	**Other**	
**VACCINE COVERAGE & VITAMIN A **(in children aged 12–23 months)						

BCG before 12 months of age	644/721	92%	86%	92%	89%	0.19
DPT-HepB3 before 12 months of age	586/721	84%	79%	88%	77%	0.24
OPV3 before 12 months of age	654/721	90%	91%	88%	90%	0.96
Measles before 12 months of age	496/721	71%	67%	65%	68%	0.61
Vitamin A in previous 6 months	499/707	74%	67%	68%	68%	0.35

**MOSQUITO NETS **(in children aged 0–23 months)						

Slept under a net last night	415/1,395	28%	28%	35%	39%	0.07
Slept under a treated net last night	159/1,395	12%	9%	10%	16%	0.19
Slept under a recently-treated net last night	125/1,395	10%	7%	6%	12%	0.19

**MALARIA AND ANAEMIA **(in children aged 0–23 months)						

Malaria parasitaemia	779/1,249	61%	62%	65%	65%	0.82
Severe anaemia (Hb < 8 g/dL)	383/1,251	25%	34%	37%	37%	0.007

**RECENT ILLNESS AND CARE-SEEKING **(in children aged 0–23 months)						

Illness in the last two weeks	673/1,404	44%	51%	58%	49%	0.07
Sought care from a Western-style health care provider (hospital, health centre, dispensary etc)	308/673	42%	47%	71%	45%	0.02
Admissions in the previous year	226/1,408	17%	15%	17%	16%	0.41

**NUTRITION**						

Exclusive breastfeeding in children under 6 months old	78/330	30%	20%	9%	21%	0.11
Underweight (weight-for-age z-score under -2) in children 0–23 months old	398/1,238	29%	37%	37%	27%	0.04

**Table 7:** Inequalities by socio-economic status in preventive care and illness in children under two years (source: household survey)

Indicator	Number of children	Percentage of children Poorest – Least Poor					Ratio Q1/Q5	P*
				
		Q1	Q2	Q3	Q4	Q5		
VACCINE COVERAGE & VITAMIN A (in children aged 12–23 months)								

BCG before 12 months of age	642/719	84	89	93	88	92	0.9	0.15
DPT-HepB3 before 12 months of age	584/719	76	79	86	75	90	0.8	0.03
OPV3 before 12 months of age	652/719	87	90	94	90	92	0.9	0.25
Measles before 12 months of age	495/719	61	68	68	71	74	0.8	0.02
Vitamin A in previous 6 months	499/705	67	64	75	72	76	0.9	0.15

MOSQUITO NETS (in children aged 0–23 months)								

Slept under a net last night	413/1,389	16	21	28	29	50	0.3	<0.0001
Slept under a treated net last night	159/1,389	4	5	11	10	25	0.2	<0.0001
Slept under a recently-treated net last night	125/1,389	3	4	6	9	20	0.2	<0.0001

MALARIA AND ANAEMIA (in children aged 0–23 months)								

Malaria parasitaemia	778/1,244	68	67	68	64	50	1.4	0.0001
Severe anaemia (Hb < 8 g/dL)	383/1,246	46	31	33	29	21	2.2	<0.0001

RECENT ILLNESS AND CARE-SEEKING (in children aged 0–23 months)								

Illness in the last two weeks	670/1,398	44	50	51	47	47	0.9	0.90
Sought care from a Western-style health care provider (hospital, health centre, dispensary etc)	307/670	44	47	45	42	49	0.9	0.81
Admissions in the previous year	226/1,402	20	13	13	18	17	1.2	0.77

NUTRITION								

Exclusive breastfeeding in children under 6 months old	77/327	30	20	33	21	17	1.8	0.09
Underweight (weight-for-age z-score under -2) in children 0–23 months old	397/1,233	43	32	30	35	25	1.7	0.001

* Test for trend

**Table 8:** Inequalities by distance from the nearest health facility in preventive care and illness in children under two years (source: household survey)

**Indicator**	**Number of children**	**Percentage of children**		**P**
			
		**Under 5 km**	**5 km and over**	
**VACCINE COVERAGE & VITAMIN A **(in children aged 12–23 months)				

BCG before 12 months of age	558/619	93	83	0.0001
DPT-HepB3 before 12 months of age	567/619	93	88	0.04
OPV3 before 12 months of age	507/619	85	76	0.02
Measles before 12 months of age	429/619	73	62	0.01
Vitamin A in previous 6 months	433/606	73	67	0.18

**MOSQUITO NETS **(in children aged 0–23 months)				

Slept under a net last night	362/1,207	33	25	0.05
Slept under a treated net last night	137/1,207	12	9	0.23
Slept under a recently-treated net last night	105/1,207	10	6	0.06

**MALARIA AND ANAEMIA **(in children aged 0–23 months)				

Malaria parasitaemia	671/1,082	60	66	0.11
Severe anaemia (Hb < 8 g/dL)	334/1,083	29	35	0.06

**RECENT ILLNESS AND CARE-SEEKING **(in children aged 0–23 months)				

Illness in the last two weeks	599/1,215	49	50	0.82
Sought care from a Western-style health care provider (hospital, health centre, dispensary etc)	277/599	51	37	0.002
Admissions in the previous year	182/1,219	15	14	0.62

**NUTRITION**				

Exclusive breastfeeding in children under 6 months old	70/290	25	23	0.69
Underweight (weight-for-age z-score under -2) in children 0–23 months old	324/1,069	29	32	0.31

We elicited information about dates of birth, sex of the child, whether they were singleton or twin births, and the date of death for any child who had died of the 7,413 women (39%) who gave birth in the three years prior to the survey, in order to estimate infant survival rates from July 2001 – June 2004. Neonatal mortality per 1000 live births was 43.2 (336/7,779) and infant mortality per 1000 live births was 76.4 (594/7,779). We also calculated infant mortality rates per 1000 per year and looked for trends in this indicator over time and differentials by other factors. Between July 2001 and June 2004 the infant mortality rate per 1000 per year was 82.5 (CI 75.6 – 90.1), and there was no evidence that it had changed over this time period (Table 9, P = 0.72). Infant mortality was almost 40% higher for teenage mothers than for older women (rate ratio (RR) 1.37, CI 1.1 – 1.7, p = 0.005), and 20% higher for mothers who had no formal education (RR 1.2, CI 1.0 – 1.4, p = 0.004). Boys and girls had similar infant mortality rates (RR 1.03, p = 0.70) and rates were also similar in male-headed and female-headed households (RR 0.93, p = 0.48). Twins had more than four times the infant mortality rate of singleton births (RR 4.59, CI 3.4 – 6.2, p < 0.0001). Neonatal mortality per 1000 child-years was more than ten times the mortality rate in children aged 1–11 months (RR = 15.1, p < 0.0001). There was some evidence of differences in infant mortality by ethnic group, with the rate in Makonde being 71.5 per 1000 CYAR and that in the Mwera and in the combined other ethnic groups being 20 to 50% higher (RR for Mwera = 1.2, RR for others = 1.5, compared with Makonde, P = 0.01). There was no evidence of inequality in infant mortality by socio-economic status (84.4/1000 child-years-at-risk (CYAR) in the poorest and 79.3/1000 CYAR in the least poor quintiles, RR = 0.94, P for trend = 0.90). However, there was evidence that infant mortality rates were higher in those living more than 5 km from the health facility compared to those living closer (95.1/1000 CYAR and 76.0/1000 CYAR respectively, RR = 1.25, P = 0.02).

**Table 9:** Differentials in infant mortality rates (source: household survey)

	Category	Deaths	Child-years	Mortality rate per 1000 child-years	RR	95% CI*	P*
Year (July–June)	2001–2	184	2,218.8	82.9	1		0.72 (for trend P = 0.75)
	2002–3	183	2,324.7	78.7	0.95	0.8 – 1.2	
	2003–4	227	2,659.8	85.3	1.03	0.8 – 1.3	
Mother's age at delivery	20–29	261	3,398.7	76.8	1		0.005
	30–39	124	1,702.2	72.8	0.95	0.8 – 1.2	
	40–49	27	364.5	74.1	0.96	0.6 – 1.4	
	Under 20	181	1,718.8	105.3	1.37	1.1 – 1.7	
Mother's education	One or more years	399	5,123.9	77.9	1		0.038
	None	194	2,063.6	94.0	1.21	1.0 – 1.4	
Sex	Girls	287	3,537.7	81.1	1		0.70
	Boys	307	3,665.5	83.8	1.03	0.9 – 1.2	
Twins	Singleton	518	6,980.1	74.2	1		<0.0001
	Twin	76	223.2	340.5	4.59	3.4 – 6.2	
Age	0–27 days	337	574.2	586.9	1		<0.0001
	28–365 days	257	6,629.0	38.8	0.07	0.06 – 0.08	
Ethnic group of household head	Makonde	220	3,075.8	71.5	1		0.01
	Mwera	246	2,867.3	85.8	1.20	1.0 – 1.4	
	Yao	29	318.7	91.0	1.27	0.8 – 2.0	
	Other	99	926.2	106.9	1.49	1.2 – 1.9	
Gender of household head	Male	478	5,695.9	83.9	1		0.48
	Female	116	1,493.2	77.7	0.93	0.7 – 1.1	
SES quintile of household	Poorest	109	1,291.1	84.4	1		0.90 (test for trend)
	Very poor	122	1,577.9	77.3	0.92	0.7 – 1.2	
	Poor	102	1,182.1	86.3	1.02	0.8 – 1.4	
	Less poor	129	1,540.9	83.7	0.99	0.8 – 1.3	
	Least poor	120	1,513.7	79.3	0.94	0.7 – 1.2	
Distance to nearest health facility	<5 km	363	4,778.9	76.0	1		0.02
	≥ 5 km	196	2,061.2	95.1	1.25	1.0 – 1.5	

**Effect of child's age on the relationship between twinning and mortality:**							

Twins under 28 days (neonates)	Singleton	280	556.6	503.0	1		<0.0001
	Twin	57	17.6	3,242.6	6.45	4.5 – 9.2	
Twins over 28 days (1–11 m)	Singleton	238	6,423.4	37.1	1		<0.0001
	Twin	19	205.6	92.4	2.49	1.5 – 4.0	

**Effect of child's age on the relationship between maternal education and mortality:**							

In neonates: Mother's education	≥ 1 year	238	407.4	584.3	1		0.93
	None	98	165.9	590.8	1.01	0.8 – 1.3	
In 1–11 month olds: Mother's education	≥ 1 year	161	4,716.7	34.1	1		0.003
	None	96	1,897.8	50.6	1.48	1.1 – 1.9	

**Effect of distance from the health facility on the relationship between maternal education and mortality**							

In those living < 5 km from a health facility: mothers education	≥ 1 year	239	3,466.	68.9	1		
	None	123	1,305.3	94.2	1.4	1.1 – 1.7	0.005
In those living ≥ 5 km from a health facility: mothers education	≥ 1 year	140	1,421.5	98.5	1		
	None	56	637.9	87.8	0.9	0.7 – 1.2	0.47

* From Poisson regression, adjusted for clustering from survey design

Of the tests for effect modification, three were statistically significant, (a) age and twinning, (b) age and mother's education, and (c) distance to the health facility and mother's education (p-value for interaction between age and twinning 0.002, between age and education 0.039, between distance and mother's education p = 0.03). These findings are explained in turn below.

Firstly, we found strong evidence that the neonatal period is particularly risky for twins, with the neonatal mortality rate for twins being over 6 times higher than that for singleton babies (RR = 6.45, CI 4.5 – 9.2, p < 0.0001). The mortality rate for twins aged 1–11 months was 2.5 times higher than that for singleton babies (RR = 2.49, CI 1.5 – 4.0, p = 0.0002).

Secondly, we found some evidence that the effect of maternal education was different in neonates and in older infants. Neonatal mortality was similar among mothers with no formal education and those who had studied (RR = 1.01, CI 0.8 – 1.3, p = 0.93), but the rate of mortality in the post-neonatal period (1–11 months of age) was about 1.5 times higher for those with no education than for mothers who had spent one or more years at school (RR = 1.48, CI 1.1 – 1.9, p = 0.003).

Thirdly, we found that the positive effect of maternal education was only apparent in families living less than 5 km from the health facility (RR = 1.4, CI 1.1 – 1.7, p = 0.005). For those living more than 5 km from the health facility there was no evidence of any association between maternal education and infant survival (RR = 0.9, CI 0.7 – 1.2, p = 0.47).

## Discussion

Most current global health efforts, including those for malaria, HIV and vaccine-preventable diseases, need to deliver in the context of rural health systems like the one described here. By including a functional and structural assessment of the health system itself, it is possible to gain insights into why some interventions work while others do not. The "staircase effect" denotes the reduction in effect at the community level of an efficacious intervention due to factors such as coverage, availability and compliance [21]. The size of the steps can differ between socio-economic groups: the poorest tend to have worse access, diagnosis, compliance and adherence, meaning that efficacious interventions do not result in equitable community effectiveness [22,23]. Delivery systems that reduce the gap between poorest and least poor are needed. The relatively short distances to health facilities, high antenatal care and good vaccine coverage suggest that peripheral health facilities have huge potential to make a difference to health and survival at the household level in rural Tanzania, even with current human resources. Nevertheless, drug shortages, staff absenteeism and water supply problems show that there is a long way to go before facilities are able to optimise the quality of care they provide.

This broad-based cross-sectional study includes both facility-based and household-based aspects of health and health care in a poor rural Tanzanian population. We had limited ability to reveal the reasons behind some of our findings. More in-depth research is needed to investigate why, for example, children born in households headed by Makonde are at lower risk of anaemia, malnutrition and infant death than those in households headed by other common ethnic groups. Although the Makonde are slightly less poor on average than other ethnic groups, the tendency for better infant survival among the Makonde was seen in all socio-economic status (SES) quintiles (data not shown). The Makonde are socially the deepest-rooted ethnic group in the area, and this may give them better social status and networks that influence well-being other than through better socio-economic status. In-depth qualitative work is ongoing that may explain what behaviours might be responsible for these findings. Furthermore, our analysis involved a large number of significance tests and thus findings should be treated with caution as it is possible that some results may be due to chance alone.

The health facility survey revealed particular problems with staff absence and drug stocks. Staff absences were common, with only about two-third of all employed staff present on the day of the survey. A group of seven essential oral treatments was found in less than half of all facilities. District-level health staff are responsible both for supportive supervision visits and drug supplies, and it seems that these aspects of their work are not prioritised. Referral is often difficult given large distances to referral centres and the lack of transport: the lack of pre-referral drugs, which may be given when referral is not possible, is of particular concern. Given the staff absences and drug shortfalls, and the fact that only about one-fifth of all facilities had a supply of clean water, it could be considered surprising that as many as 39% of women give birth in health facilities. In contrast, both childhood vaccine coverage and antenatal clinic attendance are almost universal, which means that universal coverage of preventive interventions may be an achievable goal.

We found that 38% of all women had personally experienced a child death. This is a stark illustration of how "ordinary" child deaths are in this area, as in much of sub-Saharan Africa: when a child dies, it is no great surprise. Infant and neonatal mortality rates are in keeping with findings from other sources [12,13] and although we found no evidence of a change from 2001–2004 our results are compatible with a drop in mortality around 1999–2001 [13].

In keeping with previous studies our results show that infants born to teenage mothers are at particularly high risk, as are twins, and infants born to mothers with no formal education [24,25]. More surprisingly, perhaps, we found little evidence that neonatal mortality rates were associated with maternal education, in contrast to the post-neonatal period, when mortality rates were 50% higher for mothers with no formal education compared with those who had had at least one year of schooling. In the first few days of life newborn babies and their mothers are often confined indoors at home (M Mrisho, unpublished data): it is possible that this behaviour, together with a general lack of knowledge of how to prevent newborn deaths, is the reason for similar newborn survival among babies born to mothers who had, and had not, been to school.

We found that the neonatal period is especially risky for twins, who have over six times the mortality rate of single births. A similar pattern has been reported elsewhere: in studies conducted in Nepal and Bangladesh, the neonatal mortality rate was respectively 7 times and 15 times higher than for singleton babies [26,27].

We found some evidence of disparities in vaccination coverage by socio-economic status, with ratios of coverage in the poorest to least poor of 0.8 to 0.9, as reported elsewhere [28]. We also found stark inequalities in the use of mosquito nets, parasitaemia and anaemia, similar to those found before a social marketing program for nets in another part of the country [29]. Inequalities in underweight were also in keeping with a previous survey in another part of rural Tanzania (J Schellenberg unpublished data). However, we were surprised to find no evidence of socio-economic disparities in either care-seeking for mild illness, admission to hospital, or infant survival, in contrast to findings both nationally [28] and locally [6,29-31]. This seems unlikely to be due to a lack of power as we had over 100 child deaths in each quintile. The most likely explanation is that the communities we studied are relatively homogeneous with regards to factors that influence care-seeking, such as knowledge, beliefs and means to travel.

Distance to health facilities has long been described as a barrier to their use [32-36]. In our study, children living over 5 km from a health facility had lower vaccine coverage, fewer nets, more anaemia, poorer care-seeking and higher infant mortality than those living closer. This is despite the Tanzanian public health system which reaches to village level and is relatively well used for child illness: neighbouring Uganda, for example, has roughly twice the population per health facility and care-seeking for recent childhood illness is just 8% compared with 40% in Tanzania [37,38]. The Tanzanian advantage is that three-quarters of the population live within about 5 km of their nearest facility.

## Conclusion

Observational studies such as ours reveal functional and structural aspects of the health system as well as household and community issues that might be amenable to affordable, deliverable and sustainable child survival interventions. Recent work on the mid-term assessment of the MDGs has indicated the crucial role of health systems strengthening ranging from structural over functional to educational interventions [39]. Gradually the fact that all health interventions depend on effective health-systems based delivery strategies is becoming an accepted cornerstone in public health. The present study reveals the key areas for strengthening health systems when developing and validating new disease control strategies.

## Abbreviations

BCG: Bacille Calmette-Guerrin; CI 95%: confidence interval; DPT-HepB3: Third dose of Diptheria, Pertussis, Tetanus and Hepatitis B vaccine; GPS: Global Positioning System; Hb: Haemoglobin; IPTi: Intermittent Preventive Treatment for malaria in infants; IPTp: Intermittent Preventive Treatment for malaria in pregnancy; MDG: Millennium Development Goal; OPV: Oral Polio Vaccine; ORS: Oral Rehydration Solution; PDA: Personal Digital Assistant; PPF: Procaine penicillin; SP: Sulphadoxine-Pyrimethamine; TT: Tetanus Toxoid.

## Competing interests

The authors declare that they have no competing interests.

## Authors' contributions

The study was conceived & designed by JRMAS, MM, PLA, HM, MT and DS. Substantial contributions to acquisition of the data were made by JRMAS, MM, FM, KS, CM, AKM and DS. Initial analysis and interpretation of the data was done by JRMAS, MM, KS, SCK and DS. The manuscript was drafted by JRMAS and MM. All authors were involved in critical revision for important intellectual content and approved the final version of the manuscript.

## Pre-publication history

The pre-publication history for this paper can be accessed here:


